# Will the Development of the Japanese Cancer Literacy Assessment Tool Lead to Cancer Awareness Activities with Scientific Evidence?

**DOI:** 10.31662/jmaj.2022-0149

**Published:** 2022-09-12

**Authors:** Tomio Nakayama

**Affiliations:** 1 Division of Screening Assessment and Management, National Cancer Center, Institute for Cancer Control, Tokyo, Japan

**Keywords:** cancer prevention, health literacy, health promotion

Cancer prevention and cancer screening are one of the pillars of basic plan to promote cancer control programs in Japan, but problems, such as low Human papillomavirus (HPV) vaccination rates and low cancer screening uptake rates, have not been resolved. One of the reasons for this is that correct information on cancer and cancer prevention has not been disseminated to the public people. Various educational activities have been conducted mainly by the national and local governments. In addition to the distribution of websites and leaflets on cancer prevention and cancer screening, influencers have been providing information through social networking services. Although cancer survivors, cancer patients’ family members, and health-conscious elderly people are highly interested in such information, it has not been evaluated whether the information is properly understood by the general public. Attempts to raise awareness should not be based simply on good intentions, but should be disseminated with evidence of whether or not they can increase public awareness. There have already been a significant number of studies in European Union evaluating changes in health literacy through public awareness interventions ^(1)^. For this reason, it is important to develop tools to assess public awareness of cancer to promote scientific awareness-raising activities. In this issue of the JMA journal, M. Minamitani, et al. developed a tool to assess cancer awareness among the Japanese population. Based on the EU health literacy assessment tool HLS-EU-Q47 ^(2)^, they developed a Japanese version of the JCIQ―a cancer-specific self-administered assessment tool ^(3)^. The JCIQ comprises the JCIQ-L, which is associated the literacy aspect, and the JCIQ-K, which is related to knowledge, both of which can be assessed. Generally, there are two methods for assessing health awareness: (1) to have a trained interviewer ask questions and discuss over time; (2) to use a survey instrument. The former allows for accurate evaluation, including the identification of unexpected issues, due to the large amount of information obtained. However, such methods can only be conducted with a small number of people, which can lead to subject selection bias, and the external validity of the results obtained is problematic.

Methods that use self-administered questionnaires, such as the one developed in this issue, are easy to implement and have a high affinity with the internet survey. It is highly versatile, realistic, and can be administered to numerous people, making it difficult to contaminate selection bias. It is expected to become a standard tool for surveying the public’s understanding of cancer prevention and cancer screening in the future. When conducting cancer prevention awareness activities, rather than blindly investing large sums of money, we should evaluate awareness programs using this evaluation tool in advance and invest funds only in those programs that are scientifically recognized to improve cancer literacy.

On the contrary, the JCIQ has still the problem of too many questions. The JCIQ-L and JCIQ-K have 12 and 22 questions, respectively, which is an average number of questions for a self-administered survey; however, the total of 34 questions is not expected to generate a high response rate among laypersons. When conducted as research, participants tend to be highly health-conscious and literate by nature. However, what we want to know is how many people in laypersons have low literacy and low health awareness. Because these people are less interested in the survey, the time and effort required to answer the questions should be minimized as much as possible, or they will not complete until the last question. To obtain responses from all of laypersons subjects without exception, the number of questions needs to be smaller. It is expected that a shortened version based on the JCIQ will be developed in the future.

## Article Information

### Conflicts of Interest

None
